# Strength Improvement of Glass Substrates by Using Surface Nanostructures

**DOI:** 10.1186/s11671-016-1454-1

**Published:** 2016-05-18

**Authors:** Amarendra Kumar, Kunal Kashyap, Max T. Hou, J. Andrew Yeh

**Affiliations:** Institute of Nanoengineering and Microsystems, National Tsing Hua University, No. 101, Section 2, Kuang-Fu Road, Hsinchu, 30013 Taiwan; Department of Mechanical Engineering, National United University, No. 2, Lienda, Miaoli, 36063 Taiwan; Department of Power Mechanical Engineering, National Tsing Hua University, No. 101, Section 2, Kuang-Fu Road, Hsinchu, 30013 Taiwan; Instrument Technology Research Center, National Applied Research Laboratories, 20, R&D Road VI, Hsinchu Science Park, Hsinchu, 30076 Taiwan

**Keywords:** Surface nanostructure, Bending strength, Glass, Defect density

## Abstract

Defects and heterogeneities degrade the strength of glass with different surface and subsurface properties. This study uses surface nanostructures to improve the bending strength of glass and investigates the effect of defects on three glass types. Borosilicate and aluminosilicate glasses with a higher defect density than fused silica exhibited 118 and 48 % improvement, respectively, in bending strength after surface nanostructure fabrication. Fused silica, exhibited limited strength improvement. Therefore, a 4-μm-deep square notch was fabricated to study the effect of a dominant defect in low defect density glass. The reduced bending strength of fused silica caused by artificial defect increased 65 % in the presence of 2-μm-deep nanostructures, and the fused silica regained its original strength when the nanostructures were 4 μm deep. In fragmentation tests, the fused silica specimen broke into two major portions because of the creation of artificial defects. The number of fragments increased when nanostructures were fabricated on the fused silica surface. Bending strength improvement and fragmentation test confirm the usability of this method for glasses with low defect densities when a dominant defect is present on the surface. Our findings indicate that nanostructure-based strengthening is suitable for all types of glasses irrespective of defect density, and the observed Weibull modulus enhancement confirms the reliability of this method.

## Background

Glass substrates, such as fused silica, quartz, borosilicate, aluminosilicate, and soda lime, have been widely incorporated into displays, optical elements, optoelectronic devices, and solar cells [1, 2]. However, the existence of surface or subsurface defects and inhomogeneity strongly impedes the applicability of these fragile materials [3, 4]. In the presence of surface and subsurface defects, the measured strength of glass and other brittle materials is significantly lower than the theoretical strength value [5–7]. Stress concentration at defect tips can initiate crack propagation and induce fractures in these materials [8]. Most common defects or dark spots result from handling, fixturing, chemomechanical polishing, cleaning, glazing, cutting, and dicing [9]. These defects typically appear as microcracks, affecting the strength, mechanical performance, and lifespan of glass [8, 10].

Glass weakening caused by surface defects has been an issue for decades, and many strengthening techniques are available, including altering the flaw geometry, surface compression, and thermal treatment [11]. Chemical mechanical polishing [12, 13] reduces the defect size without completely eliminating the microdefects. Thermal processes [14] minimize the defects but make post-processing machining of the glass difficult. Low-temperature chemical processes [12, 15], such as lamination or coating, fill surface cracks by depositing layers of suitable foreign materials [16–18] but modify the chemical and mechanical properties of the surface. Ion exchange processes [19] are primarily suitable for alkali-containing glasses. Coating materials containing nanoparticles reduce the stress concentration through their migration into defects, but bulk properties of substrates are lost [20]. Surface nanostructures reduce the stress at defect tips in crystalline brittle materials, such as silicon [21–23]. These structures prevent early fracture by redistributing the stress near the defect tip, which can suppress crack initiation. Crack initiation depends on the lattice structure and cleavage planes [24], which are not present in amorphous materials, such as glass. In addition, unlike silicon, glasses with different compositions have different surface and subsurface properties [25]. Bending strength improvement by surface nanostructures has no effect on bulk properties of the substrates, and higher strength is achievable without changing the current manufacturing process. Therefore, a nanostructural strengthening method should be evaluated for glass substrates, and a surface property-based analysis is required for this nanostructural strengthening method.

## Methods

Surface nanostructure strengthening depends on defect density of the glass substrates. For studying the effect of defect density on the strength improvement effect of nanostructures, three different glass compositions (fused silica, aluminosilicate, and borosilicate glasses) were selected. All three plain glass substrate surfaces were characterized by counting the surface pits using a Veeco Dimension 3100 atomic force microscope (AFM) [26]. The pit count per unit areas of the substrate surface was calculated by using AFM in combination with image analysis. The surface, subsurface, and bulk were characterized by measuring transmission wavefront using phase-stitching interferometry [27] from a Zygo verifier ATZ laser system.

The surface nanostructures were fabricated on all three glass compositions using a new technique that involves a combination of wet and dry chemical etching. Here, a 100-nm silicon layer was deposited on the glass substrate using low-pressure chemical vapor deposition. Then, the samples were dipped into a solution containing 4.6 M hydrofluoric acid (HF) and 0.02 M silver nitrate (AgNO_3_) [28] to deposit silver (Ag) nanoparticles on the silicon surface. The Ag nanoparticles acted as a mask for nanostructure formation during dry anisotropic etching by inductively coupled plasma (ICP). Inductively coupled plasma was generated by perfluorocyclobutane (C_4_F_8_) and oxygen (O_2_) gas (ratio 4:1), RF power of 100 W, ICP power of 200 W and pressure set to 13 mTorr.

The strength improvement was assessed using a three-point bending (3 PB) test for all three glass substrates. All of the samples were cut into 60 mm × 20 mm × 0.65 mm specimens using a sawing machine (Disco DAD 2H/6T) according to the ASTM 855–08 standard [29]. Each specimen was placed in a material testing machine (Hung Ta HT-2102A) that was equipped with a load cell (Hung Ta 8336) and was loaded to failure at a displacement rate of 30 mm/min using a load applicator. The bending strength ($\sigma$_*br*_) was determined using Eq. (1) [29]

1 $$ {\sigma}_{br} = \frac{1.5\ {F}_rL}{W{t}^2} $$

where *F*_r_ is the load at rupture and *L*, *W*, and *t* are the span length, width, and thickness of the sample, respectively.

Weibull analysis [30] was used to determine the reliability of the measurement, which relates the bending strength to the failure probability. The Weibull distribution can be estimated by the function shown in Eq. (2) [31].

2 $$ {P}_f\left(\sigma \right)=1-{e}^{\left\{-\kern0.5em {\left(\frac{\sigma }{\sigma_n}\right)}^m\right\}} $$

where *P*_*f*_(*σ*) is the cumulative failure probability, *σ*_*n*_ is the nominal strength, and *m* is the shape parameter (Weibull modulus).

To easily access the information, a linearized version of the equation was used, as shown in Eq. (3)

3 $$ ln\left(- \ln \left[1-{P}_f\left(\sigma \right)\right]\right)=m. ln\left(\sigma \right)-m. ln\left({\sigma}_n\right) $$

We used a high-speed camera (IDT Y-4) with illumination by a 500-W halogen lamp to record the dynamic response of the 3 PB fracture at a frame rate of 2000 frames/s with a 950-μs exposure time and 1280 × 1024 pixel resolution. The lens (TAMRON A09N) in the camera had a focal length of 30 cm.

## Result and Discussion

**Fig. 1:**
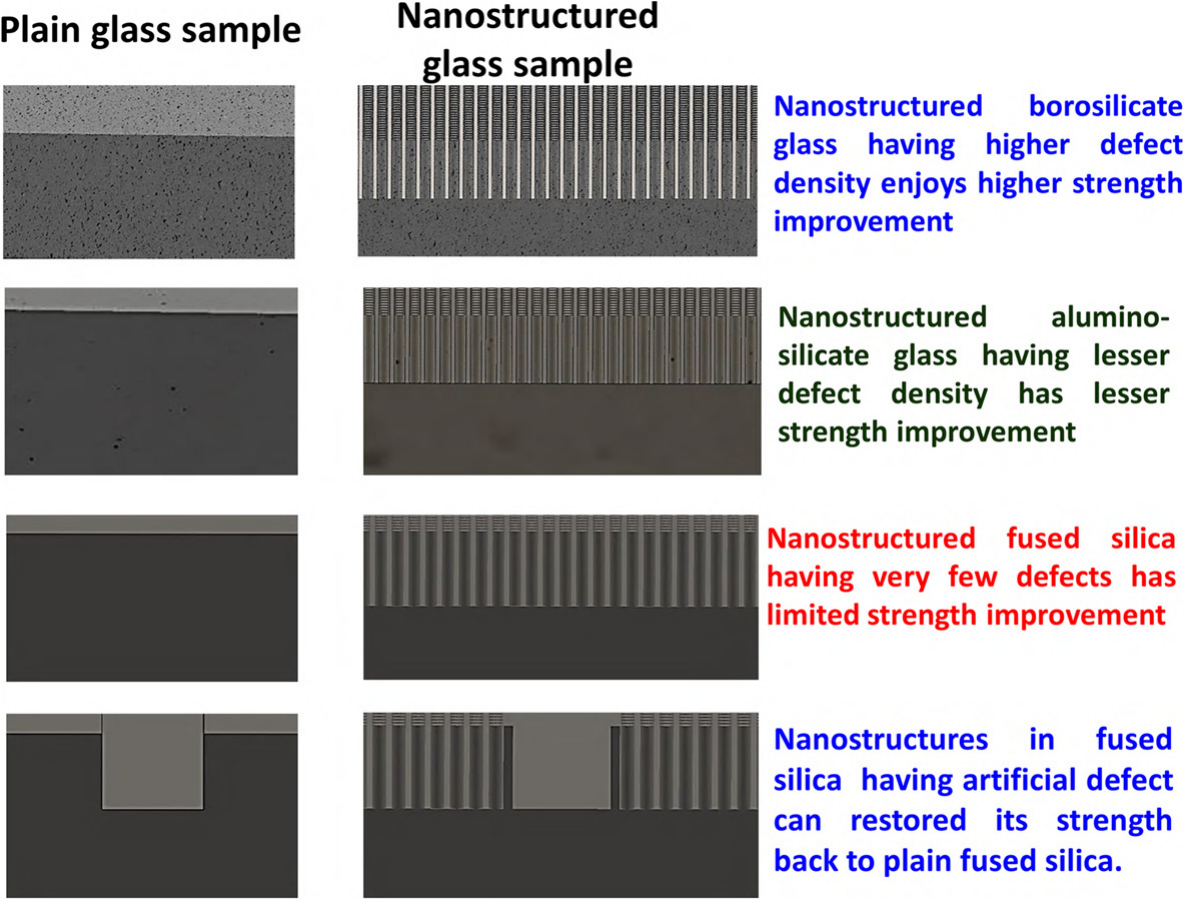
Schematic representation of the strength improvement in various glasses that exhibit different surface and subsurface properties

This study investigates nanostructure-based strength improvement of glass substrates with different compositions, which have different surface and subsurface properties. Fragile glass fails easily during bending tests, and glasses with deeper defects as well as higher defect density are more prone to failure. Nanostructure fabrication may substantially enhance the bending strength of glasses with higher defect densities by redistributing stress from the crack initiation points of random defects. However, glass substrates with lower defect densities are expected to have fewer crack initiation points and thus a lower probability of failure. This can result in less strength improvement because crack initiation points are readily available in the lower defect density glasses. The absence of a dominant stress concentration point on the glass substrates with a lower defect density may result in limited strength improvement, but these substrates are more suitable for analyzing the effect of artificial defects than are glass substrates with high-density defects. Artificial defects, which act as stress concentration points [32], substantially reduce the bending strength of these glass substrates. Nanostructure fabrication may result in the original bending strength being regained, which makes the strengthening method useful for mitigating the effects of defects formed during handling, polishing, or other post processes (Fig. 1).

**Fig. 2:**
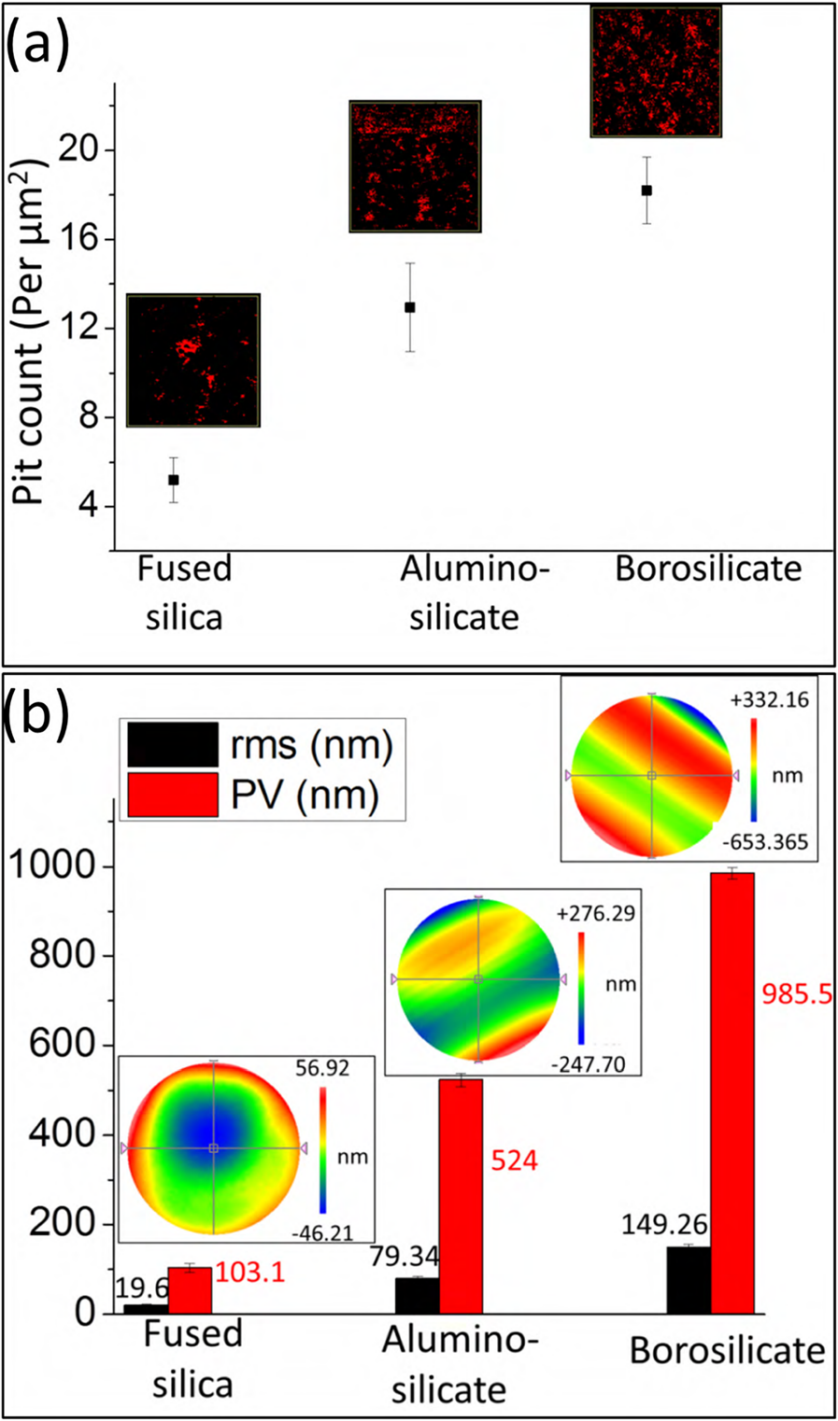
**a** Pit count per unit area measured by AFM. These values were 5.26, 12.94, and 18.2 for fused silica, aluminosilicate, and borosilicate glasses, respectively. **b** Wavefront deviation after transmission from fused silica, aluminosilicate, and borosilicate substrates. The RMS values were 19.60, 79.34, and 149.26 nm for fused silica, aluminosilicate, and borosilicate glass, respectively. Additionally, the PV values were 101.3, 524, and 985.5 nm for fused silica, aluminosilicate, and borosilicate glasses, respectively

For each substrate, the average pit count was calculated using a five-point measurement on an AFM, and scan sizes (10 μm × 10 μm) were fixed so that surface properties could be consistently compared between the different sample areas. The obtained pit count per unit area was 5.26, 12.94, and 18.2 for fused silica, aluminosilicate, and borosilicate glasses, respectively (Fig. 2a). Therefore, fused silica exhibited the lowest pit count, indicating better surface properties and a lower possibility of a stress concentration point than for the borosilicate and aluminosilicate glass substrates. The surface, subsurface, and bulk defects for the wafer can be defined as the root mean square (RMS) and peak-to-valley (PV) values of the wavefront deviation obtained using interferometry. In the absence of a glass substrate in the interferometer optical path, the RMS and PV values were 10.75 and 54.4 nm, respectively, indicating that the wavefront deviated from the transmission flat (TF). When the light wave passed through the substrate prior to being reflected by the TF, these values represent the combination of the deviation effects from the substrate and the TF. Therefore, the TF contribution was subtracted to obtain only the substrate-induced deviation effects. The RMS values were 19.60, 79.34, and 149.26 for fused silica, aluminosilicate, and borosilicate glasses, respectively. The corresponding PV values were 101.3, 524, and 985.5 nm for fused silica, aluminosilicate, and borosilicate glasses, respectively. These results demonstrate that fused silica exhibited the lowest surface and bulk defects of all three glasses (Fig. 2b).

**Fig. 3:**
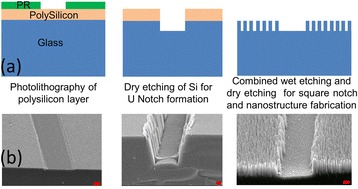
**a** Nanostructures and artificial square notch formation. **b** SEM images of masking, notch formation, and nanostructure fabrication. (All scale bars represent 500 nm)

The surface nanostructures have been previously produced using various nanofabrication methods, such as colloidal assembly [33], a sol–gel technique [34], capillary lithography [35], electron beam lithography [36], pattern transfer of natural surfaces by plasma etching [37], and electrospinning [38]. However, these methods are complex and expensive. In this study, surface nanostructures were fabricated on all three different glass compositions using combination of wet and dry chemical etching. This same fabrication methodology of wet and dry etching was implemented to form surface nanostructures and artificial defect in a single step. To form an artificial square notch, a portion of the silicon layer was removed at the desired location by conventional photolithography (Fig. 3) prior to dipping the sample into the solution. Then, the Ag nanoparticles were deposited only on the silicon surface during wet chemical etching. Rather than nanostructures, a square notch was formed on the silicon-free area during dry anisotropic etching.

**Fig. 4:**
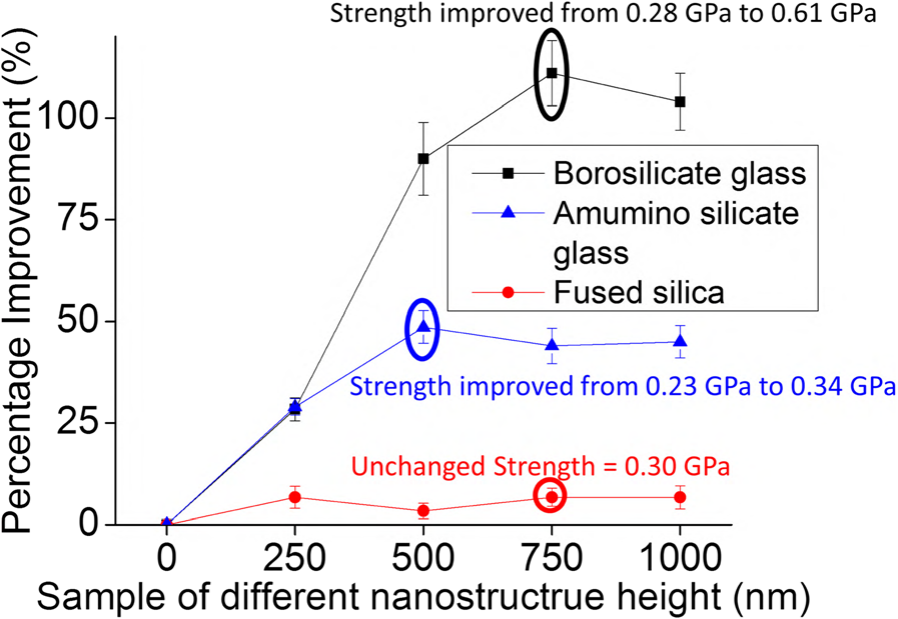
Bending strengths of the fused silica, aluminosilicate, and borosilicate glasses evaluated using a three-point bending test. The bending strengths improved by 118 and 48 % upon nanostructural formation on the borosilicate and aluminosilicate glasses, respectively

The substrate strength enhancement was evaluated using a three-point bending test for different nanostructure depths on all three glass substrates. Plain borosilicate glass exhibited a bending strength of 0.28 GPa. This strength increased to a saturation point at 0.61 GPa in the presence of 750-nm-deep nanostructures, indicating an increase of 118 % (Fig. 4). This strength improvement of 118 % in the borosilicate glass substrate is similar to the silicon substrate which showed bending strength improvement of 130 % [22]. Nanostructures can improve the strength by redistributing the stress from the defect tip to the nearby nanostructured area. This nanostructured area hinders crack initiation near the defect tip because a larger force is required to reach the same stress. Deeper nanostructures can redistribute the stress near deeper defect tips. Therefore, better results will be obtained as the nanostructure depth increases. This behavior explains the greater strength improvement for the 500-nm deep nanostructured glass than for the 250-nm-deep nanostructured glass. Saturation of the bending strength occurred at a depth where the dominant defects are not easily available. In this case, deeper nanostructures do not improve the stress redistribution, which further explains the saturation of bending strength at 750-nm-deep nanostructured borosilicate glass. The bending strength of aluminosilicate glass increased from 0.23 to 0.34 GPa in the presence of 500-nm-deep nanostructures, which represents a 48 % improvement (Fig. 4). Compared to borosilicate glass, the lower defect density, and, therefore, the fewer stress concentration points, in aluminosilicate glass (Fig. 2) limits the capacity of nanostructures to redistribute the stress. For aluminosilicate glass, the 500-nm-deep nanostructured glass exhibited a higher bending strength than that of the 250-nm-deep nanostructured glass, but saturation occurred at a depth of 500 nm. The bending strength increase was saturated with a 500-nm-deep nanostructure in aluminosilicate glass but a 750-nm-deep nanostructure in borosilicate glass because borosilicate glass has deeper defects than aluminosilicate glass. Fused silica exhibited very few stress concentration points on its surface and subsurface compared with its aluminosilicate and borosilicate counterparts. Therefore, its bending strength changed little upon nanostructure formation, which makes it suitable for analyzing the effect of the artificial defect.

**Fig. 5:**
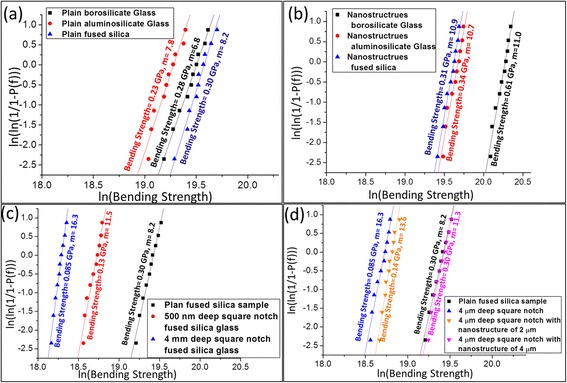
Weibull analysis before and after nanostructure fabrication. **a** Weibull moduli and bending strengths of plain fused silica, aluminosilicate, and borosilicate glasses. **b** Weibull moduli and bending strengths of fused silica, aluminosilicate, and borosilicate glasses with 750-nm-deep nanostructures. **c** Weibull moduli and bending strengths of plain deep fused silica and fused silica with 500- and 4-μm-deep square notch, respectively. **d** Weibull moduli and bending strengths of plain fused silica, unmodified fused silica with a 4-μm-deep square notch and fused silica with a 4-μm-deep square notch and 2- and 4-μm-deep nanostructures

Because of the weak impact of nanostructures on the strength of fused silica, an artificial square notch with a depth of 4 μm was fabricated in fused silica. The artificial square notch replicates the situation of defects formed during processes like handling or polishing which is a possible reason for strength reduction of any glass substrate. The bending strength of this sample decreased from 0.30 to 0.13 GPa upon addition of a 500-nm-deep square notch followed by a further decrease to 0.085 GPa in the presence of a 4-μm-deep square notch (Fig. 5c), suggesting that the artificial defect generated major stress concentration points. Upon formation of 2-μm-deep nanostructures, the bending strength improved by 65 % (from 0.085 to 0.14 GPa) for fused silica with a 4-μm-deep square notch. The artificial defect was deeper than the nanostructure and it still acted as the dominant defect. However, in comparison to plain fused silica with a 4-μm-deep artificial square notch, more force was required to generate the same stress at the notch corners. Finally, the bending strength increased to 0.30 GPa upon fabrication of 4-μm-deep nanostructures (Fig. 5d), which is in agreement with the bending strength of plain fused silica. This result indicates that an artificial defect is no longer the dominant defect. For silicon substrate, effect of the artificial defect was eliminated when the nanostructure depth was 1.5 times the artificial defect [21]. In addition, the mechanical strength reduction caused by the artificial defect was eliminated which confirms the advantage of this method for all kinds of glasses also.

The effects of the nanostructure on fused silica substrates, which have a lower defect density than the two other glass substrates and variable strength enhancement under identical test conditions, were further analyzed using Weibull analyses. Randomly distributed surface and subsurface defects cause undesirable variation in the bending strength. Under the same bending conditions, the Weibull modulus (*m*) defines a statistical variation based on the probability of failure, where a larger variation causes a lower *m*. A larger *m* value is indicative of a more predictable failure behavior, which is required for reliability considerations. This modulus was 8.2, 7.8, and 6.8 for plain fused silica, aluminosilicate, and borosilicate glasses, respectively. This result confirms the unpredictable behavior of borosilicate glass, which was caused by random defects and a larger number of random stress concentration points than in the aluminosilicate and fused silica substrates (Fig. 5a). The fabrication of 750-nm-deep nanostructured fused silica, aluminosilicate, and borosilicate glass substrates increased the Weibull modulus to 10.9, 10.7, and 11, respectively (Fig. 5b). After artificial defect formation, the estimated Weibull modulus was 8.2, 11.5, and 16.3 for plain fused silica and fused silica with 500 nm and 4-μm-deep artificial square notches, respectively. Fused silica exhibited a higher *m* value in the presence of a deeper square notch, demonstrating the dominance of this square notch over all of the other defects. Moreover, the corresponding Weibull modulus of the plain fused silica increased from 8.2 to 11.3 upon fabrication of the 4-μm-deep nanostructures. This result confirms the reliability of the nanostructure strengthening process for all three studied glass substrates.

**Fig. 6:**
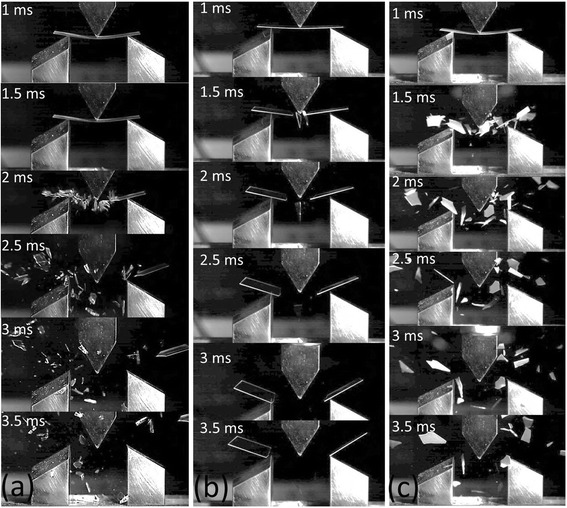
Dynamic fracture behavior of fused silica captured using a high-speed camera for fragmentation analysis. **a** Plain fused silica producing multiple fragments upon fracture. **b** Fracture of fused silica with a 4-μm-deep square notch into two major fragments. **c** Fracture of fused silica with a 4-μm-deep square notch and 4-μm-deep nanostructures into multiple fragments

Furthermore, fragmentation analysis was conducted during the 3 PB tests of the plain and nanostructured fused silica to investigate the effect of the artificial defect and nanostructure on the fracture behavior. The plain substrates broke into small fragments because of the small number of surface and subsurface defects (Fig. 6a). The artificial square notch acted as a dominant defect that overcame all of the other stress concentration points and provided major crack initiation corners that forced the sample to break into two main pieces (Fig. 6b). After surface nanostructure fabrication, the crack did not necessarily start near the notch corners because the stress near the notch corners was redistributed to other nearby nanostructure regions. The stress around the notch corners was shared by the nanostructures and led to small broken pieces because of the high strain energy absorption (Fig. 6c), [39] as observed for the plain fused silica sample.

## Conclusions

A new method for improving glass strength has been proposed and investigated based on the surface and subsurface conditions of different glass substrates. For borosilicate glasses, 750-nm-deep nanostructures enhanced the bending strength by 118 % (from 0.28 to 0.61 GPa), and 500-nm-deep nanostructures enhanced the bending strength by 48 % (from 0.23 to 0.34 GPa) for aluminosilicate glasses. In addition, these nanostructures increased the Weibull modulus of the substrates, which confirmed the usefulness of this method. The nanostructured fused silica with artificial defects that were 4-μm-deep exhibited nearly the same bending strength as plain fused silica but with a higher Weibull modulus. The number of fragments was higher during fracture, which is consistent with suppression of the artificial defect. Failure of scratch-resistant and toughened glass is very common in the presence of surface defects, and this newly developed method may improve the performance of all glass types despite different defect densities or the presence of dominant surface flaws.

## Abbreviations

3 PB: three-point bending
AFM: atomic force microscope
ICP: inductively coupled plasma
PV: peak-to-valley
RMS: root mean square
TF: transmission flat
